# Relapse of Human Chorionic Gonadotropin-Induced Hyperthyroidism and Severe Hyperemesis Gravidarum Secondary to Twin-Twin Transfusion Syndrome, With Rapid Recovery Following Fetoscopic Laser Coagulation: Case Report

**DOI:** 10.3389/fendo.2021.705567

**Published:** 2021-07-16

**Authors:** Faiza Lamine, Chiara Camponovo, David Baud, Dominique Werner, Laura Marino, Gerasimos P. Sykiotis

**Affiliations:** ^1^ Service of Endocrinology, Diabetology and Metabolism, Lausanne University Hospital and University of Lausanne, Lausanne, Switzerland; ^2^ Obstetric Service, Lausanne University Hospital and University of Lausanne, Lausanne, Switzerland; ^3^ Laboratory of Clinical Chemistry, Lausanne University Hospital and University of Lausanne, Lausanne, Switzerland

**Keywords:** twin-twin transfusion syndrome, hyperemesis gravidarum, hyperthyroidism, human chorionic gonadotropin, fetoscopic laser coagulation of placental anastomoses

## Abstract

**Background:**

Limited data have shown that, compared to uncomplicated twin pregnancies, pregnancies complicated by twin-twin transfusion syndrome (TTTS), a life-threatening condition, are associated with higher maternal serum levels of both human chorionic gonadotropin (hCG) and thyroid hormones. With the continuing expansion of assisted reproductive technologies, the rate of twin pregnancies, including those complicated by TTTS and associated hyperemesis gravidarum, is expected to increase further. Therefore, detailed descriptions of the maternal and fetal clinical outcomes of maternal thyrotoxicosis linked to TTTS can be useful for timely diagnosis and management. However, such descriptions are currently lacking in the literature.

**Case Presentation:**

We report the case of a 30-year-old woman carrying a monochorionic twin pregnancy complicated by TTTS that induced a relapse of severe hyperemesis gravidarum with overt non-autoimmune hyperthyroidism at 17 weeks of gestation. Following fetoscopic laser coagulation (FLC), both hyperemesis and hyperthyroidism improved within 1 week.

**Conclusions:**

The present experience contributes to the knowledge base on maternal thyrotoxicosis linked to TTTS and can be useful in the diagnosis and treatment of future cases; it also emphasizes the need for a high degree of clinical suspicion and for close collaboration between endocrinologists and obstetricians. Another key point is that TTTS-associated hyperemesis gravidarum and maternal hyperthyroidism should be considered in the differential diagnosis of refractory or relapsing hyperemesis gravidarum in women with monochorionic twin pregnancy, because this condition may require more stringent supportive treatment before and during the FLC procedure when the mother is overtly hyperthyroid.

## Introduction

Hyperemesis gravidarum (HG), a severe form of vomiting in early pregnancy, is a rare (0.3-1%) but potentially life-threatening condition with extreme dehydration, >5% weight loss, hypokalemia and ketosis (1). The risk of developing HG, as well as the severity of its clinical presentation, correlate with the serum levels of human chorionic gonadotropin (hCG) (1). Accordingly, HG is more frequent in twin pregnancies (1). In most cases, HG is associated with gestational transient thyrotoxicosis (GTT), a self-limited form of non-autoimmune hyperthyroidism related to the ability of hCG to bind and activate the thyroid-stimulating hormone (TSH) receptor (2–4). In uncomplicated single or twin pregnancies, both conditions are usually mild and resolve spontaneously by 16-20 weeks of gestation (WG) (4). Pregnancies complicated by twin-twin transfusion syndrome (TTTS) are associated with higher maternal serum levels of both hCG and thyroid hormones. However, there are no data regarding the maternal-fetal clinical consequences of maternal thyrotoxicosis observed in this setting.

We report here an unusual relapse of overt hCG-mediated hyperthyroidism with severe HG at 17 WG despite prior recovery from GTT by the end of the first trimester. This relapse occurred in the setting of a monochorionic (MC) twin pregnancy complicated by TTTS. Following treatment with fetoscopic laser coagulation (FLC), hyperthyroidism and TTTS improved rapidly.

## Case Presentation

**Figure 1:**
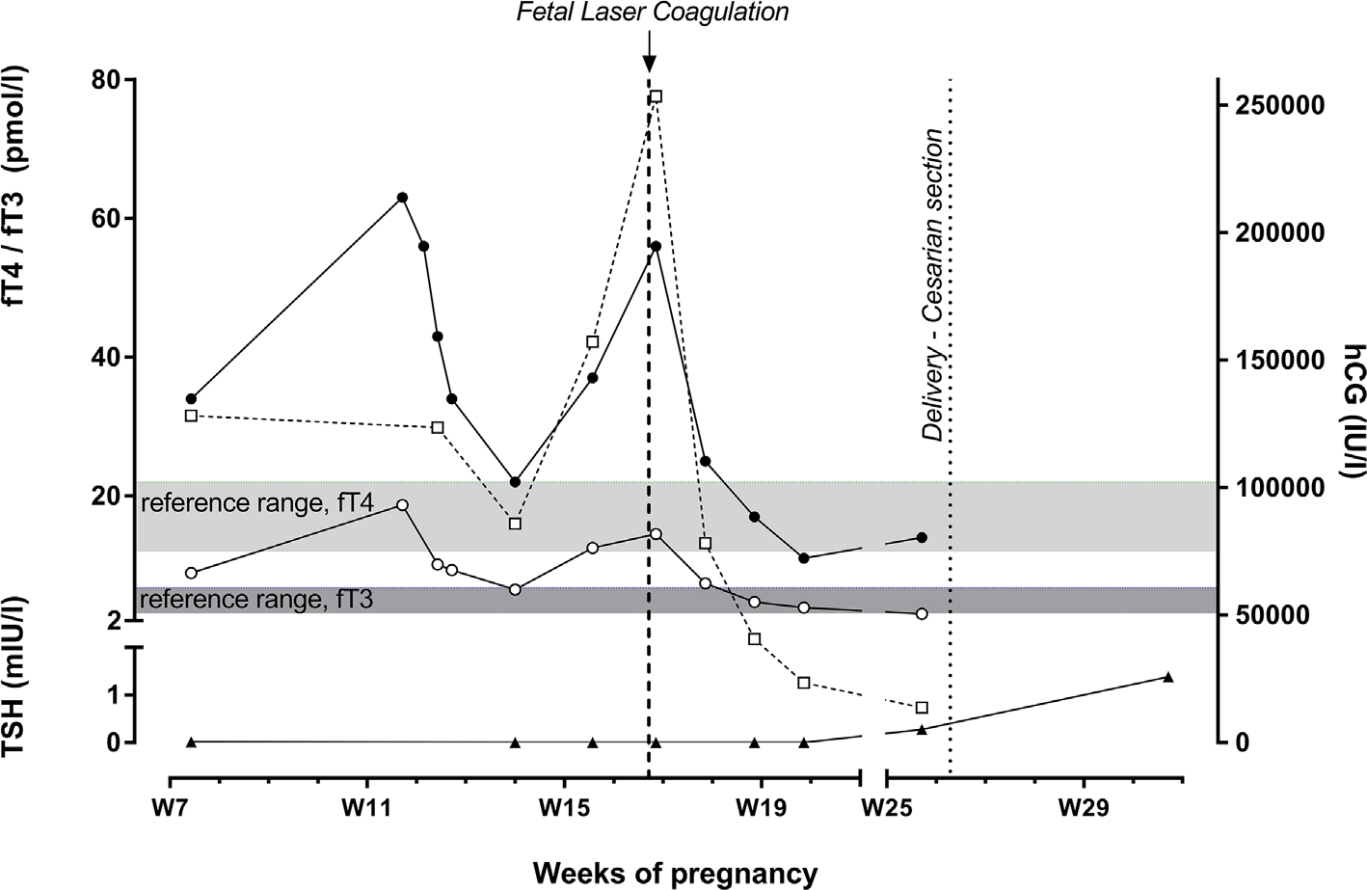
Time course of serum levels of hCG (□), TSH (▲), fT4 (●) and fT3 (○). FLC performed in the setting of TTTS led to resolution of hyperthyroidism within 1 week along with a rapid decrease in hCG levels. Hormones were measured by ECLIA (electrochemiluminescence immunoassay) on cobas e602 (Roche Diagnostics, Rotkreuz, Switzerland). Reference ranges (not trimester-specific): TSH, 0.270-4.20 mUI/l; fT4, 12-22 pmol/l; fT3, 3.1-6.8 pmol/l.

A 30-year-old woman gravida 3 para 2 carrying MC biamniotic twins was admitted to the obstetrics department at 12 WG for severe HG. She reported vomiting and nausea since 8 WG with progressive worsening despite use of oral metoclopramide. Compliance with pyridoxine supplementation previously prescribed to her was poor. She weighed 60.9 kg, having lost 5 kg over 4 weeks. Clinical examination showed hypotension (98/56 mmHg) and tachycardia (110 bpm). She had no tremor and thyroid examination was unremarkable. Abdominal ultrasound showed normal fetal status. Initial laboratory tests showed severe hypokalemia (2.4 mmol/l) with U-wave in V2-V6 on ECG. Other electrolytes, renal and liver function tests were normal. The patient was admitted to the intermediate care unit (IMCU) and was immediately started on i.v. potassium repletion, 0.9% sodium chloride and 5% dextrose infusion, i.v. thiamine 100 mg q.d., i.v. metoclopramide and rectal pyridoxine. Within 24 hours, hypokalemia had resolved and the patient was hemodynamically stable. Further investigations revealed overt hyperthyroidism (**Figure 1**). The patient had no history of thyroid disease or GTT during her previous pregnancies. Antibodies against thyroid peroxidase (TPOAb) and TSH receptor (TRAb) were negative, and thyroid ultrasonography was normal. The operational diagnosis was HG-associated GTT in the setting of a twin pregnancy. One week later, the patient was discharged with weekly follow-up at the outpatient clinic. Vomiting relief and normalization of serum levels of both free T4 (fT4) and free T3 (fT3) were observed by 14 WG.

In the setting of the monochorionic twin pregnancy, continued fetal ultrasound screening at 17 WG revealed TTTS stage 3 according to the Quintero classification (oligo-polyhydramnios sequences according to maximal vertical pockets of 1 and 10 cm in donor and recipient, respectively, absent bladder in donor and abnormal Doppler flows in both fetuses). Therefore, the patient was admitted in order to perform FLC using the Solomon technique, a minimally invasive procedure under endoscopic fetoscopy; this procedure ablates initially all visible placental vascular anastomoses, and then coagulates the entire vascular equator of the placenta to avoid recurrence of TTTS. Upon admission, the patient reported vomiting relapse since two weeks; her clinical status was consistent with severe malnutrition, and laboratory tests performed before FLC showed hypokalemia (2.6 mmol/l) with T-wave inversion in V1-V4 and hypophosphatemia (0.44 mmol/l). Supportive treatment including i.v. potassium infusion was immediately started, and FLC was successfully performed under spinal anesthesia without immediate complications. Supportive treatment was continued at the IMCU. The unexplained persistence of tachycardia (up to 120 bpm) 24 hours after FLC prompted thyroid function tests that showed overt hyperthyroidism (**Figure 1**); TPOAb and TRAb were again negative and thyroid ultrasonography was still normal. Both thyroid function tests and hCG plasma levels before FLC were retrospectively assessed on a stored plasma sample obtained at 16 WG and were clearly consistent with a relapse of overt thyrotoxicosis along with a substantial increase of hCG plasma level (**Figure 1**). Considering that hyperthyroidism was pauci-symptomatic and not related to Graves’ disease, neither beta-blockers nor antithyroid medications were prescribed. Within 1 week following FLC, vomiting had ceased and a significant drop in fT3, fT4 and hCG serum levels was observed; at 19 WG, fT3 and fT4 serum levels had normalized and the patient was discharged. She gave birth to preterm infants by emergency caesarean section at 27 WG in the setting of premature rupture of membranes for twin 1 and breech presentation for twin 2. Both preterm infants were eutrophic for gestational age. Both male twins were admitted in the neonatal intensive care unit (ICU) for respiratory distress syndrome caused by surfactant deficiency. Screening for primary congenital hypothyroidism (Güthrie card) was done at day 4 and was unremarkable in both twins. They were discharged 3 months later. In both twins, weight had reached the target for gestational age.

## Discussion and Conclusions

In this patient, the presenting features of severe HG and mild gestational thyrotoxicosis initially observed in the first trimester were attributed to the twin nature of the pregnancy. Women with uncomplicated twin pregnancies and no preexisting thyroid disease tend to have more pronounced gestational thyrotoxicosis compared to women with singleton pregnancies. This finding is consistent with the observed concurrent doubling of maternal free beta-hCG levels in twin pregnancies compared to singleton pregnancies (5). In the setting of HG, overt and symptomatic hyperthyroidism during the first trimester occurs in 46% of cases (2), and the severity of vomiting typically correlates with the degree of biochemical hyperthyroidism (2). However, gestational thyrotoxicosis typically resolves spontaneously by the end of the first trimester in both single and multiple pregnancies (5, 6).

In both single and twin pregnancies, it is unusual to observe recurrence of hCG-mediated hyperthyroidism along with exacerbation of vomiting observed nearly 2 weeks after GTT resolution with initial HG improvement and hCG decrease by the end of the first trimester. In twin pregnancies, the overall kinetic pattern of maternal hCG remains unchanged, with a peak at 10-12 WG, followed by a sustained decrease until 20 WG, and then a stabilization during the second half of pregnancy (7). In this patient, the substantial sustained increase of hCG serum level up to 250000 IU/l at 14-17 WG was apparently related to TTTS (8, 9), and it is likely to have been the cause of the subsequent HG worsening. TTTS is a severe complication of MC twin pregnancies occurring in nearly 10% of cases. It is characterized by the development of abnormal arteriovenous placental anastomoses that result in uncompensated unidirectional blood flow from a “donor” to a “recipient” twin. If untreated, severe TTTS is associated with up to 90% perinatal mortality and severe morbidity (10). Screening with serial ultrasonography every 2 weeks starting at 16 WG is therefore recommended in the setting of MC twin pregnancies (10).

The limited literature (one published study available) on maternal thyroid function in the setting of TTTS suggests that TTTS could be considered a cause of hCG-induced maternal hyperthyroidism (9). However, to the best of our knowledge, there are no data describing the clinical symptoms and outcomes in women presenting altered thyroid function tests associated with TTTS. The novelty of the present case report is that we described the clinical features associated with serum fT3, fT4 and hCG kinetics before and following TTTS treatment in an individual pregnant woman. This could be helpful to guide the timely diagnosis and safe management of this particular form of maternal hyperthyroidism during pregnancy. Unlike the transient nature of GTT, TTTS-associated maternal hyperthyroidism, such as observed in the present case, is *a priori* not a self-limited condition and it is not expected to resolve until FLC is performed. Moreover, in the present case, the recurrence of overt hyperthyroidism went undiagnosed before FLC.

TTTS is associated with a significant increase of maternal hCG levels as compared to uncomplicated twin pregnancies in the mid-gestational period (8). In 131 pregnant women diagnosed with TTTS, median concentration of serum hCG between 16-26 WG was 96952 IU/l (interquartile range, 50964-155472), which was 5.4 times the median value in singleton pregnancies (9). Only 4 patients (out of 131, 3%) had hCG levels above 250000 IU/l, as observed in our case. The most likely mechanisms are the large placental volume and placental hypoxia secondary to uteroplacental blood flow disruption by the polyhydramnios surrounding the recipient fetus (8). Successful treatment of TTTS with FLC of placental vascular anastomoses led to decrease and then normalization of hCG levels by 2 and 4 weeks, respectively (11). The observations in the present case are consistent with these data. Furthermore, in the present patient, hCG decrease was associated with resolution of severe vomiting 1 week following FLC.

Regarding maternal thyroid function in the setting of TTTS, moderate positive correlations were reported between levels of maternal serum hCG and both fT4 (R=0.33, p<0.001) and fT3 (R=0.22, p=0.030). Nearly half of the patients had fT3 levels above the upper limit of the normal range. Even though fT3 levels were above the upper limit of the reference range in nearly 50% of women, clinical features were not described in the subgroup of women presenting with biochemically overt hyperthyroidism (9). In most cases, both fT4 and fT3 levels decreased concurrently with the decrease of hCG levels at 2 and 4 weeks after successful FLC (9). The same pattern was also observed in the present patient; clinical symptoms of thyrotoxicosis were mild, and supportive therapy, such as beta-blockers, was not needed.

Because hyperthyroidism was not diagnosed before the procedure, the occurrence of thyroid storm in the setting of hCG-mediated hyperthyroidism could have been a serious perioperative complication, as has been reported for molar pregnancies, which are also forms of hCG-induced hyperthyroidism (12, 13).

Further prospective longitudinal studies are needed on maternal thyroid function in the setting of MC pregnancies complicated by TTTS, to assess the prevalence and clinical relevance of hCG-mediated hyperthyroidism in this setting. Endocrinologists and obstetricians should consider TTTS-associated maternal hyperthyroidism in the differential diagnosis of patients with relapsing or refractory hyperemesis gravidarum because this condition is not expected to be self-limited and to resolve until FLC is performed, and it may require a more stringent supportive treatment when the mother is overtly hyperthyroid.

## Data Availability Statement

The original contributions presented in the study are included in the article/supplementary material. Further inquiries can be directed to the corresponding author.

## Ethics Statement

Written informed consent was obtained from the individual(s) for the publication of any potentially identifiable images or data included in this article. Under Swiss law, case reports of under 5 cases are exempt from ethics approval.

## Author Contributions

FL, CC, DB, DW, LM, and GPS analyzed and interpreted the patient data. FL, CC, and GPS drafted the manuscript. All authors contributed to the article and approved the submitted version.

## Funding

GPS was supported by a 2016 Leenaards Foundation Fellowship for Academic Promotion in Clinical Medicine. The funder provided salary support to secure protected research time for GPS. The funder had no direct involvement in the preparation of the present paper.

## Conflict of Interest

The authors declare that the research was conducted in the absence of any commercial or financial relationships that could be construed as a potential conflict of interest.

## Abbreviations

ECLIA, electrochemiluminescence immunoassay; FLC, fetoscopic laser coagulation; fT3, free T3; fT4, free T4; GTT, gestational transient thyrotoxicosis; hCG, human chorionic gonadotropin; HG, hyperemesis gravidarum; MC, monochorionic; TPOAb, thyroid peroxidase antibodies; TRAb, TSH receptor antibodies; TSH, thyroid-stimulating hormone; TTTS, twin-twin transfusion syndrome; WG, weeks of gestation.
